# Supplementary material for: Prevalence and associated factors of visual impairment among adult diabetic patients visiting Adare General Hospital, Hawassa, South Ethiopia, 2022
Source: PLoS One. 2022 Oct 13;17(10):e0276194. doi: 10.1371/journal.pone.0276194 (PMC9560493; doi:10.1371/journal.pone.0276194)
Supplement: Attachment — Submitted filename: Reviewer respoonse.docx [file pone.0276194.s001.docx]

**Point by point response to reviewer`s comment**

**Manuscript title: Prevalence and Associated Factors of Visual Impairment Among Adult Diabetic Patients Visiting Adare General Hospital, Hawassa, South Ethiopia, 2022**

**Manuscript number:**

**Dear Reviewer.** Thank you for providing us the chance to revise our manuscript. We are great full for your careful and in-depth reading of the manuscript. We appreciate your thoughtful comments and constructive suggestions, which help us to improve the quality of the manuscript. Having this we addressed all the concerns raised and incorporated our reflections in the revised manuscript. We attempted to address all the issues raised to be corrected.

Many thanks

**Responses to Reviewers**

**Reviewer- 1**

1. **Reviewer’s comment:** Limitation of the study is also represented by the lack of data about 2 major risk factors that have been associated to diabetic retinopathy, that are albuminuric chronic kidney disease and high HDL levels, which should be added to the appropriate section.

**Authors’ response:** Thank you for your comment. We accept the comment and made necessary correction in the revised manuscript.

2. **Reviewer’s comment:** Discussion should be treated more in depth. For example, the role played by physical activity in ameliorating insulin resistance. In fact, a wrong lifestyle may produce an excessively positive caloric balance, thus causing insulin resistance by enhancing visceral adipose tissue and consequently releasing a much higher level of free fatty acids, TNF-α, adipokines and hyperglycaemia which will lead to increased inflammation, endothelial disfunction and, as a consequence to increased prevalence of diabetes complications and in particular retinopathy (doi: 10.3390/antiox10020270).

**Authors’ response:** Thank you for your depth insight. We accept it and made incorporated this relevant information with in the revised manuscript.

**Reviewer - 2**

1. **Reviewer’s comment**: Abstract: There are some typing errors.

**Authors’ response**: Thank you for your comment. We accept the comment and made necessary correction in the revised manuscript

2. **Reviewer’s comment:** Introduction: “Diabetes is a complex…” seems too generic, I would suggest to specify which type of diabetes.

**Authors’ response:** Thank you for your suggestion. We accept it and we tried to address your suggestion in the revised manuscript

But diabetes both type I and II were found resulted hyperglycemia with different mechanism.

3. **Reviewer’s comment** - Please insert the entire words when an acronym is used for the first time throughout the text

**Author response**: Thank you for your comment. We made correction within the revised manuscript

4. **Reviewer’s comment**: Methods - The authors write “systemic co-morbidities were collected from each patient's medical chart”. I would suggest to briefly specify how co-morbidities diagnosis has been done, particularly for what concerns the statistically associated peripheral neuropathy.

**Author response:** Thank for your comment. We accept the insight and include the clinical diagnostic criteria of peripheral neuropathy in the revised manuscript. Even though here were the diagnosis criteria of other systemic co morbidities included in our study Diagnosis of hypertension was made based on patients’ blood pressure when a person's systolic blood pressure is ≥140 mm Hg and/or their diastolic blood pressure ≥90 mm Hg following repeated examination. Anemia was diagnosed based on laboratory results of blood hemoglobin concentration (below 130 g/L for men, 120 g/L for females). Heart disease and nephropathy were diagnosed by their respective clinics, and patients were already on their medication. Peripheral neuropathy was diagnosed based on a person’s medical history and physical examination (inspection of feet, [vibration perception](https://www.sciencedirect.com/topics/medicine-and-dentistry/vibration-sense), [ankle reflex](https://www.sciencedirect.com/topics/medicine-and-dentistry/achilles-reflex) test, and monofilament test)

5. **Reviewer’s comment**: Discussion: The authors write “Studying the prevalence in this setup would increase the magnitude of visual impairment.” Recently, some authors have demonstrated that telemedicine could allow to implement the screening programs also during a pandemic period (Galiero R, Pafundi PC, Nevola R, et al. The Importance of Telemedicine during COVID-19 Pandemic: A Focus on Diabetic Retinopathy. J Diabetes Res. 2020;2020:9036847. Published 2020 Oct 14. doi:10.1155/2020/9036847; Sasso FC, Pafundi PC, Gelso A, et al. Telemedicine for screening diabetic retinopathy: The NO BLIND Italian multicenter study. Diabetes Metab Res Rev. 2019;35(3):e3113. doi:10.1002/dmrr.3113). I would suggest to briefly discuss this point.

**Author response:** Thank you for your suggestion. We accept it and we tried to show the impact of Telemedicine for screening diabetic retinopathy on reduction of visual impairment among patients with diabetes**.**

6. **Reviewer’s comment**: Limitations - This is a single-center study, thus it seems difficult to fully generalize the results. I would suggest adding this issue to limitations section.

- To evaluate glycemic control, the study lacks glycated hemoglobin, I would suggest to discuss this issue or to move in to limitation section.

**Authors’ response:** Thank you for your comment. We accept the comment and made necessary correction in the new revised manuscript.

7. **Reviewer’s comment:** Conclusion:
   - The authors write “Diabetic patients should engage in regular physical activity for at least 150 minutes per week, control their glucose levels with proper use of medication, and have regular eye exams.”, however no references have been associated with this sentence. I would suggest to at least a reference linked to this point

**Author response:** Thank you for your critical insight. But in this study, poor physical exercise, poor glucose control and having no history of eye examination were significantly associated with visual impairment. Based on this fining, we have recommend that those patient with diabetes should engage in regular physical activity for at least 150 minutes per week, control their glucose levels with proper use of medication, and have regular eye exams. So, we believed that no need of referencing.
